# Aboveground-belowground biodiversity linkages differ in early and late successional temperate forests

**DOI:** 10.1038/srep12234

**Published:** 2015-07-17

**Authors:** Hui Li, Xugao Wang, Chao Liang, Zhanqing Hao, Lisha Zhou, Sam Ma, Xiaobin Li, Shan Yang, Fei Yao, Yong Jiang

**Affiliations:** 1 State Key Laboratory of Forest and Soil Ecology, Institute of Applied Ecology, Chinese Academy of Sciences, Shenyang 110164, China; 2 Great Lakes Bioenergy Research Center, University of Wisconsin, Madison 53706, USA; 3 State Key Laboratory of Genetic Resources and Evolution, Computational Biology and Medical Ecology Lab, Kunming Institute of Zoology, Chinese Academy of Sciences, Kunming 650223, China

## Abstract

Understanding ecological linkages between above- and below-ground biota is critical for deepening our knowledge on the maintenance and stability of ecosystem processes. Nevertheless, direct comparisons of plant-microbe diversity at the community level remain scarce due to the knowledge gap between microbial ecology and plant ecology. We compared the α- and β- diversities of plant and soil bacterial communities in two temperate forests that represented early and late successional stages. We documented different patterns of aboveground-belowground diversity relationships in these forests. We observed no linkage between plant and bacterial α-diversity in the early successional forest, and even a negative correlation in the late successional forest, indicating that high bacterial α-diversity is not always linked to high plant α-diversity. Beta-diversity coupling was only found at the late successional stage, while in the early successional forest, the bacterial β-diversity was closely correlated with soil property distances. Additionally, we showed that the dominant competitive tree species in the late successional forest may play key roles in driving forest succession by shaping the soil bacterial community in the early successional stage. This study sheds new light on the potential aboveground-belowground linkage in natural ecosystems, which may help us understand the mechanisms that drive ecosystem succession.

Understanding ecological linkages between the aboveground and belowground biota is an important challenge for our knowledge on the maintenance and stability of ecosystem processes^1^. Soil microorganisms play fundamental roles in biogeochemical cycling and are major drivers of terrestrial ecosystem diversity and productivity, and hence, the relationship between soil microbial community and aboveground attributes (plant productivity, composition, and diversity) has attracted greater attention than ever before^2^,^3^,^4^. Nevertheless, direct comparisons of plant-microbial community diversity remain limited due to the knowledge gap between microbiology and general ecology. Such a knowledge gap is likely a consequence of the methodological difficulties associated with observing microbes in nature and a product of the very different historical paths followed by the disciplines of microbiology and general ecology^5^,^6^. Macro-ecologists have traditionally followed more theoretical and holistic paths, and microbial ecologists have often relied on more reductionist experimental approaches^5^. Accordingly, the aboveground and belowground communities have usually been surveyed independently, and paired information on plant and microbial communities collected within the same sampling sites is limited.

Plants (producers) provide the organic carbon required by the decomposer subsystem, and the decomposer subsystem in turn breaks down dead plant material and indirectly regulates plant growth and community composition by determining the supply of available soil nutrients^1^. Given the predominant functional associations between plants and soil microbes, high aboveground diversity is usually proposed to be linked to high belowground diversity because of diverse litter types and root exudates^1^,^7^. Nevertheless, the few studies comparing plant-microbial diversity have reported mixed results, and a variety of aboveground-belowground relationships have been proposed^8^. Laboratory-based experiments have demonstrated the positive plant-microbial diversity relationships between specific plant taxa and the root-associated microbial groups, such as arbuscular mycorrhizal fungi (AMF)^9^,^10^,^11^. It was also reported that the symbiotic, nitrogen-fixing bacteria rhizobia promoted the evenness of plant communities^12^. On the contrary, the uncoupling of plant and microbial diversity was reported at the BioCON grassland field experiment^13^, in which higher AMF spore richness was observed in the lower plant species richness treatment. Additionally, a negative correlation between plant and ammonia-oxidizing bacterial diversity was found in grasslands at different successional stages^14^.

In addition to the α-diversity comparison, our understanding of the association of plant-microbial β-diversity is quite limited. A latest publication^4^ compared the diversity of plant, bacterial, archaeal and fungal communities across temperate grasslands worldwide, and highlighted that plant diversity predicts the beta- but not the alpha-diversity of soil microbes. The systematic comparison of β-diversity between belowground microbes and aboveground plants has rarely been documented in natural forest ecosystem. To the best of our knowledge, only one published study directly compared the aboveground-belowground β-diversity in the western Amazon rainforest in Peru^15^. These authors concluded that the fungal community structure was strongly linked to plants by comparing seven selected plots with exact corresponding aboveground and belowground community information.

The diversity and community composition of a forest stand may not be sufficiently captured by tree species richness and abundance *per se*. Productivity diversity, which can be approximately estimated by the composition of tree size, should not be neglected. It was presumed that belowground diversity is not only correlated with tree species diversity but also with tree size diversity because more resources aboveground may sustain a larger and possibly more diverse soil and litter community^16^. The positive correlation between the diversity of host-specific microbial groups, such as AMF^3^,^9^,^17^ and nitrogen-fixing bacteria^12^,^18^, and overall plant productivity was previously reported in grassland ecosystems. However, no previous studies have considered the species-specific “productivity diversity” when dealing with the plant-bacteria relationship.

Owing to the conflicting results in the plant-microbe α-diversity relationship and the limited number of studies of aboveground-belowground β-diversity connections in natural forest ecosystems, we would like to more directly address the following questions in the present study: Is high belowground (α-, β-) diversity necessarily correlated with high aboveground diversity? Are these patterns consistent in different successional stages in natural temperate forest ecosystems? Do key species or groups above or below the ground contribute to shaping the community on the other side of the surface? To answer these questions, we selected two types of temperate natural forests located in the Changbai Nature Reserve, Northeast China, representing early (~80 years) and late (~300 years) successional forests, and compared the plant-bacteria diversity in large-scale by collecting a large number of samples. Further, we attempted to determine whether the abundance or productivity of specific tree species contributed to shaping the belowground bacterial communities.

## Results

### Soil properties in the early and late successional forests

The soil physico-chemical properties of the two forest types were similar in soil texture, SOC, H^+^, Al^+^, and CEC (Mann-Whitney U test *P* > 0.05). The soils from the late successional forest (BLKP) contained higher TN, TK and TS, but had lower soil pH, C/N ratios and TP compared with the early successional forest (PB) (Table 1). However, the soil property dissimilarity among the sampling sites within each forest type, calculated using the Euclidean distance, showed no significant difference (homogeneity of multivariate dispersion test on average distance-to-centroids, *F* = 0.014, *P* = 0.9063) between the two forests, with an average value of 3.739 (s.d. = 1.721, range = 0.316 ~ 11.55) for the BLKP forest and 3.778 (s.d. = 1.735, range = 0.310 ~ 12.12) for the PB forest.

### Alpha-diversities of plant and bacterial communities are not positively linked

**Figure 1:**
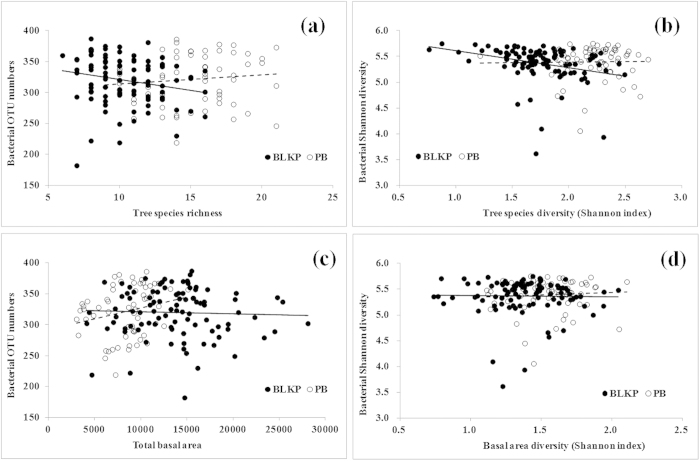
Correlations between plant and bacterial α-diversities. We regressed bacterial observed OTUs against **(a)** tree species richness and **(c)** total basal area, and bacterial Shannon diversity against **(b)** tree species Shannon diversity and **(d)** basal area Shannon index.

The observed bacterial OTU numbers in the BLKP forest (mean = 320, s.d. = 39, range = 182 ~ 387) and PB forest (mean = 321, s.d. = 41, range = 219 ~ 386) were very similar (Mann-Whitney U test *P* > 0.05). However, we found a higher (Mann-Whitney U test *P* < 0.05) tree species richness in the PB forest (mean = 15.24, s.d. = 3.10, range = 9 ~ 21) compared with the BLKP forest (mean = 10.37, s.d. = 2.33, range = 6 ~ 16). A regular linear regression showed that the observed bacterial OTU number in the PB forest exhibited a positive but non-significant correlation with the aboveground plant species richness (*r* = 0.129, *P* = 0.321). However, the exact reverse pattern was found in the BLKP forest, where bacterial richness was negatively correlated with tree species richness (*r* = −0.276, *P* = 0.007; Fig. 1a). Observed Chao 1 estimates of bacterial richness showed a comparable pattern, which was not significant, for all detections (Supplementary Fig. S1a).

The same results were observed for the Shannon index. There was no significant difference in the bacterial Shannon indices between the two forests (Mann-Whitney U test *P* > 0.05), with an average value of 5.365 (s.d. = 0.353, range = 3.616 ~ 5.750) for the BLKP forest and 5.395 (s.d. = 0.330, range = 4.056 ~ 5.753) for the PB forest. However, significant differences in the plant Shannon diversity index were observed between the two forests (Mann-Whitney U test, *P* < 0.001); the PB forest was associated with a higher plant Shannon index. The correlations of the Shannon index between the above- and below-ground communities also showed trends that were similar to species richness (Fig. 1b). The bacterial Shannon diversity had a significant negative correlation with the plant Shannon index in the BLKP forest (*r* = −0.423, *P* < 0.001), but no significant correlation was detected in the PB forest (*r* = 0.028, *P* = 0.832).

We also examined the tree-size effects on the soil bacterial diversity. First, we regressed the total basal area of all the trees within each plot against the bacterial OTU numbers, and found no significant correlation (*P* > 0.05) between the total basal area and the bacterial OTU richness in the BLKP forest (Fig. 1c). Nevertheless, a significant positive correlation between the bacterial richness and total basal area was found in the PB forest, estimated either by observed OTU numbers (*r* = 0.286, *P* = 0.025, Fig. 1c) or Chao 1 estimates (*r* = 0.327, *P* < 0.05; Supplementary Fig. S1b). The Shannon index of the tree basal area, calculated based on species-specific basal area, had no significant (*P* > 0.05) correlation with the bacterial Shannon diversity in either forest (Fig. 1d).

The Spearman’s rank correlation coefficients between the α-diversity indices and the measured soil characteristics indicated that the bacterial and plant α-diversity were each influenced by different soil variables, and there was no consistency between the two forests. The bacterial OTU number and the Shannon index in the BLKP forest were negatively correlated with TP (*r* = −0.342 and −0.314, respectively, *P* < 0.05 for both) and CEC (*r* = −0.219 and −0.235, respectively, *P* < 0.05 for both). However, plant species richness in the BLKP forest was significantly (*P* < 0.05) influenced by a variety of edaphic properties, including TN (*r* = 0.243), the C/N ratio (*r* = −0.353), TP (*r* = 0.212), TK (*r* = −0.285), TS (*r* = 0.211) and CEC (*r* = 0.221). The Shannon index of the tree species diversity was negatively correlated with the C/N ratio (*r* = −0.209, *P* < 0.05) and TK (*r* = −0.330, *P* < 0.05). The sum of the total basal area within each plot was positively correlated with the C/N ratio (*r* = 0.234, *P* < 0.05) but negatively correlated with TK (*r* = −0.299, *P* < 0.05). In the PB forest, the measured edaphic properties showed no signifcant influence on almost all of the bacterial and plant *α*-diversity indices. For more details, see Supplementary Table S2.

### Bacterial β-diversity is coupled with plant β-diversity in the late stage of succession but is closely correlated with soil property distances in the early successional forest

The α-diversity that we computed above followed the narrow-sense definition of community diversity. To systematically reveal the aboveground-belowground diversity linkage, the β-diversity of the bacterial and plant communities were also compared in this study. The bacterial β-diversity was significantly higher in the late successional (BKLP) forest than in the early successional (PB) forest (multivariate dispersion test, *F* = 8.18, *P* = 0.0048). The average Bray-Curtis distance of the bacterial community was 0.908 (s.d. = 0.024, range = 0.827 ~ 0.982) in the BLKP forest, and 0.896 (s.d. = 0.021, range = 0.827 ~ 0.963) in the PB forest. Strikingly, the dissimilarity of the bacterial community in the BLKP forest was significantly influenced by the aboveground tree species richness (Mantel *r* = 0.148, *P* = 0.007). Nevertheless, the bacterial β-diversity in the PB forest was not affected by the tree species richness (Mantel *r* = −0.039, *P* = 0.728).

**Figure 2:**
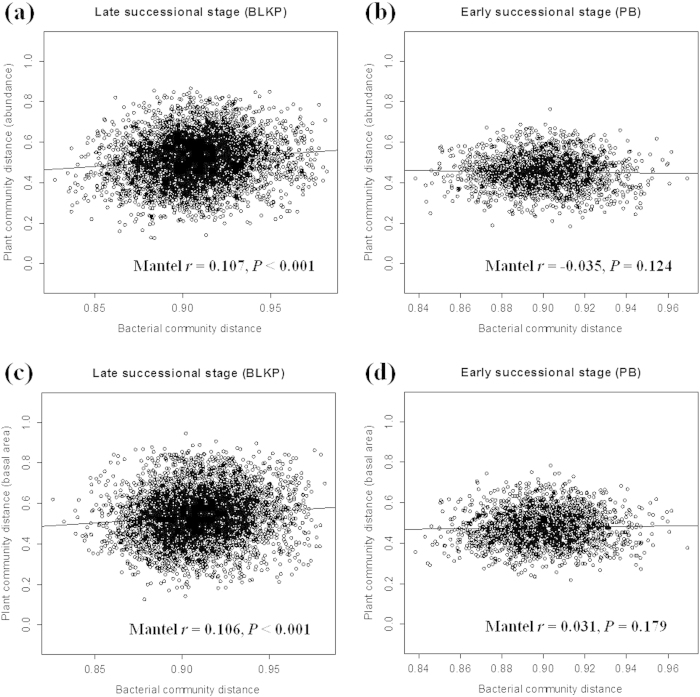
Comparison of plant and bacterial β-diversity in two temperate forests with different successional stage in Changbai Mountain, China. Bray-Curtis distances of plant community were computed based on tree species abundance (**a**) and (**b**); and basal area of each tree species (**c**) and (**d**).

Overall, the aboveground β-diversity was much lower than that of the belowground bacterial communities, in terms of either the tree species abundance dissimilarity or the basal area distance matrix. Plant β-diversity computed based on tree species abundance varied greatly across the BLKP forest with mean value of 0.514 (s.d. = 0.118, range = 0.126 ~ 0.866). The plant community β-diversity measured using the Bray-Curtis distance in the PB forest (mean = 0.453, s.d. = 0.084, range = 0.183 ~ 0.763) was significantly lower than that in the BLKP forest (multivariate dispersion test, *F* = 13.69, *P* = 0.0030), with a lower variation across selected plots. We observed a strong coupling of the plant and bacterial β-diversity in the late successional (BLKP) forest (*r* = 0.107, *P* < 0.001; Fig. 2a); however, the resemblance of plant communities in the early successional stage (PB) had no connection with the belowground community dissimilarities (*r* = −0.035, *P* = 0.124; Fig. 2b). The Jaccard distances of the plant and bacterial communities were also used for comparison of the above- and below-ground β-diversity, and the same patterns were detected (Supplementary Fig. S2).

The plant community distances prepared from the basal area of each tree species showed a pattern that was similar to that calculated from the tree species abundance. The BLKP plots exhibited a significantly (multivariate dispersion test, *F* = 11.09, *P* = 0.0011) higher plant β-diversity (mean = 0.537, s.d. = 0.126, range = 0.126 ~ 0.948) compared with the PB forest (mean = 0.478, s.d. = 0.089, range = 0.216 ~ 0.784). A regression between the bacterial β-diversity and the tree basal-area distances also revealed a positive correlation in the BLKP forest (*r* = 0.106, *P* < 0.001; Fig. 2c), indicative of biotic coupling. However, the non-significant correlations in the PB forest (*r* = 0.031, *P* = 0.179; Fig. 2d) show that similar plant communities are not necessarily associated with similar soil bacterial communities.

Considering the important role of the soil mass in linking the plant community and belowground biota, we further tested the influence of the soil properties on the plant and bacterial communities using partial Mantel test. Although we observed no significant differences in the average soil property dissimilarity between the BLKP and PB forests, the soil-bacteria relationship was quite different. A significant correlation between the bacterial β-diversity and the soil dissimilarity was found in the PB forest when we removed the effects of the plant community distances, calculated based either on tree species abundance (partial Mantel *r* = 0.177, *P* = 0.020) or on basal area (partial Mantel *r* = 0.172, *P* = 0.019). In contrast, there was no effect of the soil property distance on the bacterial community dissimilarity in the late successional (BLKP) forest (Table 2). When the bacterial community distance was controlled as a constant, no significant relationship was found between the plant community and the soil variables in either forest type (Supplementary Table S3), indicating that the plant community distance was not associated with similarity in the environmental characteristics of the selected temperate forests.

### Effects of specific tree species on shaping the bacterial community composition

**Figure 3:**
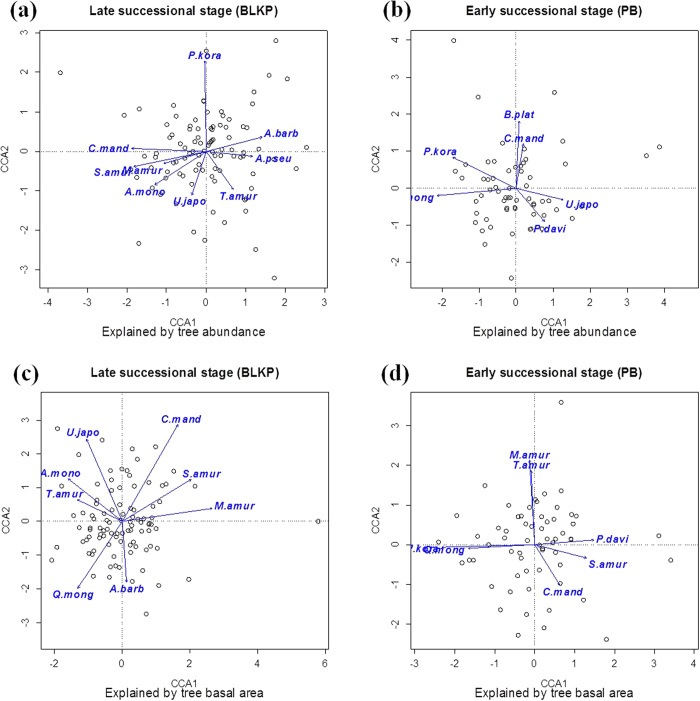
Ordination plots of canonical correspondence analysis (CCA) to explore the relationship between bacterial communities and selected aboveground dominant tree species (vectors) in the early and late successional forests. Ordinations explained by tree species abundance (**a**) and (**b**) and by tree basal area (**c**) and (**d**) were shown respectively, and only significant correlations with *P* < 0.05 were fitted on the ordination. Abbreviation: *S.amur: Syringa amurensis; A.barb: Acer barbinerve; P.kora: Pinus koraiensis; A.pseu: Acer pseudosieboldianum; C.mand: Corylus mandshurica; A.mono: Ace mono; T.amur: Tilia amurensis; U.japo: Ulmus japonica; M.amur: Maackia amurensis; Q.mong: Quercus mongolica; B.plat: Betula platyphylla; P.davi: Populus davidiana*.

To determine whether key aboveground tree species contribute to maintaining the community structure on the other side of the surface, we performed a canonical correspondence analysis (CCA) on the bacterial communities and treated the abundance or the basal area of the dominant tree species as constrained variables. The CCA biplot clearly showed the relationship between the abundance/tree-size of important tree species and the bacterial community structure (Fig. 3). In the early successional forest, the abundance of 6 dominant trees showed significant (*P* < 0.05) correlations with the ordination scores, including 5 canopy and sub-canopy trees (*Quercus mongolica, Pinus koraiensis, Betula platyphylla, Ulmus japonica*, and *Populus davidiana*) and an understory species (*Corylus mandshurica*). *Pinus koraiensis* and *Quercus mongolica* were shown as the top two important trees with correlations R^2^ > 0.3. With respect to the tree-size, 7 tree species were revealed as significant variables in explaining the variations in the bacterial communities, including 5 canopy and sub-canopy tree species (*Pinus koraiensis, Maackia amurensis, Tilia amurensis, Quercus mongolica* and *Populus davidiana*), and two understory species (*Corylus mandshurica* and *Syringa amurensis*). The basal area of *Pinus koraiensis* and *Maackia amurensis* were the two most important vectors associated with the first and second axis, respectively. In the late successional forest, the abundance of *Pinus koraiensis* was shown to be the most important variable for shaping the soil bacterial community (R^2^ = 0.372, *P* < 0.001). The ordination plot also indicated that *Corylus mandshurica* and *Syringa amurensis* were another two important tree species related to the patterns of the bacterial communities, with respect to abundance (R^2^ = 0.250 and 0.246, respectively, *P* < 0.001 for both) as well as basal area (R^2^ = 0.382 and 0.199, respectively, *P* < 0.001 for both).

The above results indicated that the abundance and productivity of specific tree species in temperate forests might play a fundamental role in driving the belowground bacterial community. We also detected the relationship between the plant communities and the relative abundance of dominant bacterial phyla using the same method, i.e., a CCA analysis for the plant community with the relative abundance of the dominant bacterial phyla as the constrained variables. The selected bacterial taxa included Proteobacteria, Acidobacteria, Verrucomicrobia, Actinobacteria, Planctomycetes, Bacteroidetes, Firmicutes, and Gemmatimonadetes, which were detected as the dominant phyla in these forest soils (Supplementary Fig. S3). Although we found some significant correlations (data not shown), we did not derive any convincing relationships from the correlations, due to the large-scale diversity of the bacterial communities in the soil.

## Discussion

It has often been hypothesized that high diversity in plant species can result in increased diversity of soil microbes because the resource heterogeneity (diverse litter types and root exudates) is proposed to be associated with a greater diversity of decomposers and detritivores^19^,^20^. Nevertheless, our results do not completely support this general hypothesis. We observed no obvious aboveground-belowground diversity linkage in the early successional forest, in terms of both the α-diversity and the β-diversity. In the late successional forest, we detected a negative α-diversity correlation, suggesting that high belowground α-diversity is not always associated with high aboveground α-diversity. Additionally, a positive β-diversity correlation was found in the later stage of succession, indicating that the high plant compositional dissimilarity between the two sampling sites was coupled with the high bacterial community distance.

We documented clearly different patterns of the plant-bacteria diversity relationship in the early and late successional forests, which may have been caused by the differences in the ecosystem characteristics of the two successional stages. The early stage of succession (PB forest) was usually characterized by low ecosystem stability, high plant species diversity, low productivity (represented by total basal area), and rapid soil nutrient cycling^21^. In the late stage of succession, however, all ecosystem attributes showed the reverse pattern. Plant species diversity usually increases during early succession as new species arrive, but declines in later succession as competition eliminates opportunistic species and leads to dominance by local competitors^22^. The productivity of a late successional forest is much higher than that of a natural secondary forest, creating a resource-rich but physically stable macro-environment.

In our study, we observed a negative plant-bacteria α-diversity correlation in the late successional forest. The plant diversity was much lower at the later stage of succession, and did not increase with an increase in productivity (Supplementary Fig. S4). On the other hand, the bacterial diversity was maintained at a high level, which may be caused by the rich resources provided by the high aboveground productivity and the functional redundancy of bacterial communities^23^. The asynchronous variation in the above- and below-ground communities during succession could be a plausible explanation for the negative correlation in α-diversity between the two sides. Although significant plant-bacteria α-diversity correlation was not observed in the early stage, we still found a weak positive pattern. The positive plant-bacteria α-diversity relationship in the earlier stages may be induced by the increasing aboveground diversity, and thus, diverse litter types and root exudates. Based on the facts described above, we suggest that the aboveground-belowground α-diversity relationship will change over time, from positive to negative, during the long-term succession of forest ecosystems. However, the selection of only two successional stages does not provide sufficient information to predict the aboveground-belowground diversity relationship along an ecosystem succession. In the present study, we attempted to select another natural forest in the same area, with a similar environmental background but at a different successional stage. Regretfully, we could not find such an ideal forest without human disturbance. To get more comprehensive knowledge on the full spectrum of above- and below-ground diversity relationship along succssional process, future field studies should be conducted.

Differences in the plant-bacteria β-diversity relationship indicate successional changes from a disturbed to an established ecosystem. The early stage of succession is usually considered as a disturbed and highly dynamic ecosystem. The soil nutrients are mainly stored in the litter and soil^21^ and are consumed rapidly; this is associated with rapid biogeochemical cycling. Accordingly, the bacterial β-diversity responded more to the soil dissimilarity matrix than to the plant community distances in the early successional stage (Table 2). On the contrary, a significant positive effect of plant β-diversity on the bacterial community dissimilarities was observed in the late successional stage, representing an established and highly stable ecosystem. In the late stage of succession, there is an equilibrium between the energy used from sunlight and the energy released by decomposition, and between the uptake of nutrients from the soil and the return of nutrients to the soil by litter fall. The soil nutrient resource is stored mainly in the litter and living biomass, and biogeochemical cycling is very slow^21^. Therefore, the current inputs of carbon are not important in determining the variation in the soil properties, which could be the primary cause of the lack of correlation between the bacterial community dissimilarities and the soil property distances (Table 2). Instead, the bacterial β-diversity was governed by the aboveground community dissimilarities, measured by either tree species abundance or tree basal area, in the late successional forest. Considering that the differences in the plant community may also be driven by edaphic factors, it is not easy to determine whether bacterial communities were responding primarily to the plant community, the common soil environment, or both. Nevertheless, neither the plant nor the bacterial community was restricted by local variation in soil properties in the late successional forest (Table 2 and Supplementary Table S3). Consequently, it seems that the correlation between the plant and bacterial community structure is driven, at least partially, by the direct effects of the plants on the bacterial community structure. The differences in aboveground-belowground diversity relationship between the early and late successional forests suggest that different mechanisms are responsible for structuring diversity in these forest ecosystems.

We determined that competitive tree species may drive ecosystem succession by shaping the soil bacterial community in the early successional stage. In the earlier stage of succession, the plant communities are usually dominated by fast-growing, well-dispersed species (r-selected life-histories), represented by *Betula platyphylla, Populus davidiana*, and *Quercus mongolica* in our present study. As the ecosystems undergo succession, these species tend to be replaced by more competitive (k-selected) species, such as *Pinus koraiensis*. Because plant species differ in both the quantity and quality of the resources that they return to the soil, individual plant species may have an important effect on the components of the soil biota and the processes that they regulate^24^. Competitive plant species are frequently more important for soil microorganisms than intolerant ones; thus, it was not surprising that the abundance of *Pinus koraiensis*, the dominant competitor in the BLKP forest, contributed more to shaping the bacterial communities at the late successional stage. Interestingly, *Pinus koraiensis* was also detected as the most important variable in explaining the variations in the bacterial communities in the early successional forest, both in terms of abundance and tree-size. After 80-year clear-cutting or fire of the BLKP forest, the PB forest was formed by natural succession. At this stage in the succession, *Pinus koraiensis* had an average abundance of 10.24 and an average basal area of 73.88 cm^2^ within the 20-m × 20-m plots (Table S1). This indicated that, even as saplings, *Pinus koraiensis* have already begun to play a significant role in explaining the variations in the bacterial communities in the early successional stage. Thus, we propose that the competitive tree species, which became the dominant competitors in late succession, may play a key role in ecosystem succession by determining the soil bacterial community in the early successional stage. However, this hypothesis needs to be further examined with more direct empirical evidence.

In addition to *Pinus koraiensis*, the broad-leaved tree species *Quercus mongolica, Maackia amurensis, Tilia amurensis* and *Ulmus japonica* also made a considerable contribution to the bacterial community variation, in both the early and the late successional stages, which may be caused by the fact that they are the important constructive species in the temperate forests of Northeast Asia. The shrub species *Corylus mandshurica* and *Syringa amurensis* are widely distributed in early and late successional forests, and exert significant effects on bacterial communities. It was suggested that the shrub layer plays a very important role in promoting nutrient cycling in the forest^25^ and is thus linked tightly to belowground communities. As the pioneer species, *Betula platyphylla* and *Populus davidiana* were likely the driving factors for shaping the bacterial community in the early successional forest, but these two species gradually disappeared during the succession, and were eventually replaced by other shade-tolerant species in the absence of disturbances. Conversely, *Acer momo* and *Acer barbinerve* made more contributions to the development of the bacterial communities in the late successional forest compared with the early stage. It was reported that these two trees are relatively more shade-tolerant and become superior competitors during succession^26^. Clearly, there are species that may act as both pioneer and tolerant ones, depending on the circumstances.

In view of the above aspects, three major conclusions were reached. First, high belowground bacterial diversity is not necessarily correlated with high aboveground plant diversity in temperate forests of Northeastern Asia, and second, the relationship depends on the successional stage of the forest ecosystem. We observed a negative plant-bacteria α-diversity correlation in the late stage of succession, but no obvious trend was found in the early successional stage. The bacterial β-diversity was significantly determined by the aboveground species richness and was strongly coupled with the plant β-diversity in the late stage of succession, estimated either by plant abundance or by tree basal area. In the early successional forest, however, the bacterial β-diversity was not associated with aboveground plant β-diversity, but was closely correlated with soil property distances. Third, the dominant competitive tree species might play a key role in ecosystem succession by influencing the soil bacterial community in earlier successional stages.

Our study has contributed a systematic documentation on both plant and bacterial communities in typical temperate forests in Northeast Asia and uniquely fills the knowledge gap associated with the aboveground-belowground diversity relationship in natural forest ecosystems. Nevertheless, the measures of bacterial community do not represent the whole belowground biota, particularly at those sites with low pH where fungi can be the most active members of the community. In future work, the plant-fungal diversity relationships deserve further consideration regarding the evaluation of aboveground-belowground relationship patterns. Additionally, beyond the two stages of early and late, there should be other stages that had not been considered in this study. Therefore, with current data we have, our conclusions obtained in this study can only address the two relatively distinct stages we defined in this study. Nevertheless, given that our study is the first exploration of such topic, we believe that our finding should be instructive for designing future studies.

## Methods

### Site description and soil sampling

The study area is part of the Changbai Nature Reserve, which is located along the border of China and North Korea (41°43–42°26′N; 127°42′–128°17′E). The Reserve has been protected from logging and other severe human disturbances since it was established in 1960, and joined the World Biosphere Reserve Network under the UNESCO Man and the Biosphere Programme in 1980. The study site has a typical temperate continental monsoon climate with long cold winters and warm summers. The vegetation on Changbai Mountain represents the typical types of natural forest in Northeast Asia^26^,^27^,^28^.

Two forest types, Broad-leaved Korean pine mixed forest and Poplar-Birch forest, representing late (~300 years) and early (~80 years) successional stages, respectively, were chosen in this study. The Broad-leaved Korean pine (BLKP) mixed forest is the most common vegetation type with respect to species composition and ecosystem structure in this region. The dominant tree species in the BLKP include *Tilia amurensis*, *Pinus koraiensis*, *Acer mono*, *Corylus mandshurica*, *Acer pseudo-sieboldianum*, and *Quercus mongolica*. The Poplar-Birch (PB) forest was formed by natural succession after clear-cutting or burning of the BLKP forest, and represents the early successional stage of the BLKP forest. The dominant tree species in the PB forest are *Betula platyphylla*, *Populus davidiana*, *Syringa amurensis*, *Quercus mongolica*, *Tilia amurensis*, *Acer mono*, and *Pinus koraiensis*. The dominant tree species in these two forests are listed in order of the important value of tree species.

The selected forests are located at the core zone of the Changbai Nature Reserve. A total of 95 and 61 representative plots, each 20 m × 20 m in size, were selected in the BLKP and PB forests, respectively. To record the accurate geo-distance of the soil sampling sites, soil samples were collected from the geometric center of each plot. At the center point, two soil cores were collected within a few centimeters of each other and homogenized together to form an independent sample. Each soil core was collected using a 5-cm diameter PVC core to a depth of 10 cm after the fresh forest floor litter had been removed. Each soil sample was placed in a plastic bag and transported to the laboratory on ice. Within 48 h of sampling, field-moist samples were sieved through a 4-mm sieve and were thoroughly homogenized and subdivided into two subsamples. The one subsample for determining soil properties was stored at 4 °C, and the other for DNA extraction was maintained at −80 °C.

The edaphic properties were analyzed using the methods described previously^29^. Briefly, soil pH was measured in a soil slurry with a 2.5:1 water: soil ratio using a glass electrode. Total organic carbon (TOC) was determined using a TOC analyzer (Analytikjena HT1300, Germany) after removing soil carbonates with 1 M HCl. Total nitrogen (TN) and total sulfur (TS) was determined using an elemental analyzer (Perkin-Elmer 2400II CHN elemental analyzer, USA). Total phosphorus (TP) was measured using the Mo-Sb Anti spectrophotometric method, and total potassium (TK) was detected using a flame atomic absorption spectrophotometer (AA6800, Shimadzu, Japan). Cation exchange capacity (CEC) was measured using the ammonium acetate method. The soil C/N ratio was calculated using the TOC and TN datasets. Particle size fractions were determined using hydrometer methods.

### Documentation of aboveground plant communities

Within each 20-m × 20-m plot, we surveyed and recorded the trees and shrubs that were at least 1 cm in diameter at breast height (DBH, 1.3 m above ground) and summarized tree species richness, abundance and basal area. All plant data were surveyed at the time we collected the soil samples^27^.

### Characterization of bacterial communities with 454 pyrosequencing

Soil genomic DNA was extracted from approximately 0.7 g of moist soil using the high salt SDS-based method^29^. The crude DNA was purified using chloroform/isoamyl-alcohol (24:1) and precipitated with 0.6 volume of isopropanol. The variable region V_1_-V_3_ of the 16S rRNA gene was amplified using the primers 27F and 534R^30^, which included 454 Life Science’s A or B sequencing adapters, respectively. The 534R primer included an 8-bp barcode for multiplexing of samples during sequencing. The amplicons amplified with one set of barcoded primers were pooled at equimolar concentrations and pyrosequenced at the Institute for Bioinformatics and Evolutionary Studies (IBEST) (University of Idaho, Moscow, ID, USA) on a Roche FLX 454 automated pyrosequencer running the Titanium chemistry.

The raw sequencing data were processed mainly using Mothur v1.12.2^31^. Briefly, low-quality sequences (those with sequences <200 bp in length with an average quality score of <25) were removed from the datasets, and the qualified sequences were parsed into individual samples according to the specific barcode. Chimera detection was performed using Uchime^32^ against recommended databases. The representative sequences were then identified and aligned against version 108 of the Silva SSU 16S rRNA database^33^. Because diversity is unavoidably correlated with the number of sequences collected, each sample was rarefied to an equal number of 520 sequences. OTU clustering was carried out using Mothur with a 0.03 cutoff, which was used to characterize the biodiversity of the bacterial population at the species level. For taxonomy analysis, the unique sequences were assigned to phyla by the RDP-II classifier using a 50% confidence threshold^34^. The sequences obtained in this study were uploaded and made available at the NCBI SRA under the accession number SRP028799 (Biosample numbers SAMN02222467 ~ SAMN02222628).

### Diversity estimates

The bacterial community was defined as all phylotypes (OTUs identified by a 3% distance level) originating from an individual soil sample, and the plant community was defined as the tree species identified in a single quadrat. For bacterial α-diversity, the observed OTU numbers and Chao1 richness estimator were used to estimate the species richness in the sampling assemblage. The bacterial diversity within each individual sample, which constituted richness and species abundance, was estimated using the non-parametric Shannon’s diversity index. The richness and diversity within the plant community was quantified as the total number of tree species and Shannon diversity. We chose Shannon’s index because it reflects both evenness and richness of species, without favoring either dominant or rare species.

For β-diversity, we used two methods to measure the bacterial community dissimilarity: the Bray-Curtis and Jaccard distance matrices. Because the two methods provided similar results, here, we only report the results based on the Bray-Curtis distances; the results for the Jaccard distances are provided in the Supplementary materials online. The Bray-Curtis and Jaccard distance matrices were also computed for the plant community by using the information of the documented tree and shrubs species. To examine the effects of tree-size β-diversity, the Bray-Curtis distance matrix was constructed based on the basal area of each tree species.

### Statistical analyses

We used linear regression models to test the relationship between plant and bacterial α-diversity (richness and Shannon diversity). Spearman’s rank correlations were used to evaluate the correlation between the α-diversity indices and soil properties, and the correlation coefficients and significance values were calculated using the SPSS package (SPSS Inc., v 17.0, Chicago, Illinois). To compare the dataset between the two forest types, Mann-Whitney U tests were performed in SPSS.

To investigate whether the aboveground plant species richness influenced the belowground β-diversity, the Bray-Curtis distances of the bacterial community were correlated with the corresponding differences between the tree species richness using the Mantel test (Spearman’s rank correlation) implemented in the R statistical environment. To link the aboveground-belowground β-diversity, the bacterial community distances were regressed against the plant community dissimilarities (estimated by the tree species abundance or tree basal area). Partial Mantel tests were then used to determine the effects of soil properties on the plant and bacterial communities by holding one distance matrix constant and vice versa. Because most soil variables are correlated, the soil similarity was calculated as the Euclidean distance generated from the scaled principal component analysis scores for each site based on 13 soil variables, including pH, TOC, TN, the C/N ratio, TP, TK, TS, H^+^, Al^3+^, CEC, % Sand, % Silt and % Clay. To compare the differences in the average β-diversity between the two forests, a multivariate dispersion test was performed using the betadisper function in the Vegan^35^ package in R.

To determine whether some dominant tree species contributed to shaping the soil bacterial community belowground, the dominant species of the canopy, sub-canopy and understory layers from the two forests were selected and treated as the environmental variables in the bacterial community canonical correspondence analysis (CCA). The selected trees and shrub species are listed in Supplementary Table S1. The plant species abundance data were log (X + 1) transformed, and the basal area data were square root transformed. The key tree species were fitted onto the ordination using the envfit function of the Vegan^35^ package in R with 999 permutations.

## Additional Information

**How to cite this article**: Li, H. *et al*. Aboveground-belowground biodiversity linkages differ in early and late successional temperate forests. *Sci. Rep*. **5**, 12234; doi: 10.1038/srep12234 (2015).

## Supplementary Material

Supplementary Information

## Figures and Tables

**Table 1:** Soil physico-chemical characteristics of the two forests. Shown are the mean values, and the values in parentheses are standard deviations (n = 95 for BLKP forest, and n = 61 for PB forest).

Forest type	SOC (g kg^−1^)	TN (g kg^−1^)	C/N	TP (g kg^−1^)	TK (g kg^−1^)	TS (g kg^−1^)	Soil texture %			CEC cmol(+) kg^−1^	H^+^ (mmol kg^−1^)	Al^+^ (mmol kg^−1^)	pH
							Sand	Silt	Clay				
BLKP	69.72 (18.54)	7.66 (2.05)	9.52 (3.51)	0.67 (0.29)	14.65 (1.04)	0.86 (0.47)	20.31 (5.83)	52.30 (6.40)	27.40 (3.57)	33.57 (6.17)	1.81 (1.94)	8.34 (9.85)	5.25 (0.21)
PB	74.31 (27.00)	7.06 (2.34)	10.98(3.38)	1.08 (0.38)	13.75 (1.66)	0.61 (0.22)	20.26 (8.07)	51.73 (6.63)	28.01 (5.68)	32.42 (7.45)	1.61 (1.20)	8.19 (8.24)	5.42 (0.22)
Mann-Whitney U test *P* value	0.346	**0.032**	**<0.001**	**<0.001**	**0.001**	**0.001**	0.436	0.928	0.825	0.235	0.146	0.876	**<0.001**

**Table 2:** The influence of soil properties on bacterial community composition determined by Mantel test with the plant community distance matrix partialed out.

Forest	Tree species abundance distance matrix partialed out		Tree basal area distance matrix partialed out	
	*r*	*P*	*r*	*P*
BLKP	−0.011	0.562	−0.010	0.561
PB	**0.177**	**0.020**	**0.172**	**0.019**
