# Supplementary material for: Aboveground-belowground biodiversity linkages differ in early and late successional temperate forests
Source: Sci Rep. 2015 Jul 17;5:12234. doi: 10.1038/srep12234 (PMC4505317; doi:10.1038/srep12234)
Supplement: Supplementary Information [file srep12234-s1.doc]

**Aboveground-belowground biodiversity linkages differ in early and late successional temperate forests**

Hui Li^1^, Xugao Wang^1^, Chao Liang^1, 2^, Zhanqing Hao^1^, Lisha Zhou^1^, Sam Ma^3^, Xiaobin Li^1^, Shan Yang^1^, Fei Yao^1^, Yong Jiang^1*^

^1^State Key Laboratory of Forest and Soil Ecology, Institute of Applied Ecology, Chinese Academy of Sciences, Shenyang 110164, China; ^2^Great Lakes Bioenergy Research Center, University of Wisconsin, Madison 53706, USA; ^3^State Key Laboratory of Genetic Resources and Evolution, Computational Biology and Medical Ecology Lab, Kunming Institute of Zoology，Chinese Academy of Sciences, Kunming 650223, China

**Supplementary Table S1 | Selected tree species in bacterial community canonical correspondence analysis (CCA).** The average number and basal area of individuals in each 20m×20m plot are shown (mean ± SD).

| Forest type | Vertical layer | Tree species | Average number of individuals | Average basal area |
| --- | --- | --- | --- | --- |
| Late successional forest (BLKP) | Canopy layer | *Pinus koraiensis* | 3.19 | 3407.49 |
|  |  | *Tilia amurensis* | 4.29 | 3572.58 |
|  |  | *Quercus mongolica* | 1.05 | 1550.64 |
|  |  | *Ulmus japonica* | 1.6 | 653.01 |
|  |  | *Fraxinus mandschurica* | 0.96 | 2228.61 |
|  | Sub-canopy layer | *Acer mono* | 7.8 | 843.91 |
|  |  | *Maackia amurensis* | 0.86 | 117.74 |
|  |  | *Acer pseudosieboldianum* | 6.99 | 294.90 |
|  | Shrub layer | *Corylus mandshurica* | 20.05 | 52.88 |
|  |  | *Syringa amurensis* | 4.28 | 55.10 |
|  |  | *Acer barbinerve* | 14.83 | 51.58 |
| Early successional forest (PB) | Canopy layer | *Betula platyphylla* | 11.37 | 3480.23 |
|  |  | *Populus davidiana* | 4.48 | 2219.13 |
|  |  | *Quercus mongolica* | 5.84 | 629.98 |
|  |  | *Ulmus japonica* | 3.11 | 110.37 |
|  |  | *Fraxinus mandschurica* | 1.66 | 307.54 |
|  | Sub-canopy layer | *Pinus koraiensis* | 10.24 | 73.88 |
|  |  | *Tilia amurensis* | 8.27 | 424.39 |
|  |  | *Acer mono* | 9.39 | 269.90 |
|  |  | *Maackia amurensis* | 4.23 | 291.07 |
|  | Shrub layer | *Corylus mandshurica* | 11.56 | 31.29 |
|  |  | *Syring amurensis* | 20.66 | 151.37 |

**Supplementary Table S2 | Correlation between plant / bacterial α-diversity and selected soil properties.**

| Forest type | Diversity index | SOC | TN | CNR | TP | TK | TS | Sand | clay | silt | CEC | pH | H^+^ | Al^3+^ |
| --- | --- | --- | --- | --- | --- | --- | --- | --- | --- | --- | --- | --- | --- | --- |
| Late successional forest (BLKP) | Bacterial OTUs No. | NS* | NS | NS | **-0.342** | NS | NS | NS | NS | NS | -0.219 | NS | NS | NS |
|  | Bacterial Shannon *H*’ | NS | -0.216 | NS | -0.314 | NS | NS | NS | NS | NS | -0.235 | NS | NS | NS |
|  | Tree species richness | NS | 0.243 | **-0.353** | 0.212 | **-0.285** | 0.211 | NS | NS | NS | 0.221 | NS | NS | NS |
|  | Tree Shannon *H*’ | NS | NS | -0.209 | NS | **-0.330** | NS | NS | NS | NS | NS | NS | NS | NS |
|  | Total basal area | NS | NS | 0.234 | NS | **-0.299** | NS | NS | NS | NS | NS | NS | NS | 0.214 |
|  | Basal area Shannon *H*’ | NS | NS | NS | NS | NS | NS | NS | NS | NS | NS | NS | NS | NS |
| Early successional forest (PB) | Bacterial OTUs No. | NS | NS | NS | NS | NS | NS | NS | NS | NS | NS | NS | NS | NS |
|  | Bacterial Shannon *H*’ | NS | NS | NS | NS | NS | NS | NS | NS | NS | NS | NS | NS | NS |
|  | Tree species richness | NS | NS | NS | NS | NS | NS | NS | NS | NS | NS | NS | NS | NS |
|  | Tree Shannon *H*’ | NS | NS | NS | NS | NS | NS | NS | NS | NS | NS | NS | NS | NS |
|  | Total basal area | NS | NS | NS | NS | NS | NS | -0.261 | NS | NS | NS | NS | NS | NS |
|  | Basal area Shannon *H*’ | NS | NS | NS | NS | NS | NS | NS | NS | 0.287 | NS | NS | NS | NS |

* NS= non significant (*P* > 0.05). **Bold numbers: *P* < 0.001.

**Supplementary Table S3 | The influence of soil properties on plant community composition (calculated based on tree species abundance or basal area) determined by partial mantel test with the bacterial community distance matrix partialed out.**

| Forest | Partial Mantel test between soil and tree species abundance distance matrix | | Partial Mantel test between soil and tree basal area distance matrix | |
| --- | --- | --- | --- | --- |
|  | *r* | *P* | *r* | *P* |
| BLKP | 0.036 | 0.259 | 0.026 | 0.310 |
| PB | 0.094 | 0.084 | 0.079 | 0.149 |

**Supplementary Figure S1 | Correlation between bacterial Chao 1 estimators and (a) tree species richness and (b) total basal area.** Bacterial Chao 1 estimator showed a positive correlation with tree species richness in early successional (PB) forest, but a reverse pattern was observed in late successsinal (BLKP) forest, non-significant (*P* > 0.05) in both cases. Bacterial Chao 1 estimator was positively correlated with total basal area in early successional forest (*r* = 0.327, *P* < 0.05), but no obvious trend was found in late successsinal forest.

**Supplementary Figure S2 | Regression of plant and bacterial β-diversity based on Jaccard distance matrices.** **(a)** In late successional forest, we observed a strong coupling of plant and bacterial communities (Mantel *r* = 0.085, *P* < 0.001). **(b)** In early successional forest, no significant correlation was found between the pant and bacterial communites (Mantel *r* = 0.012, *P* = 0.595).


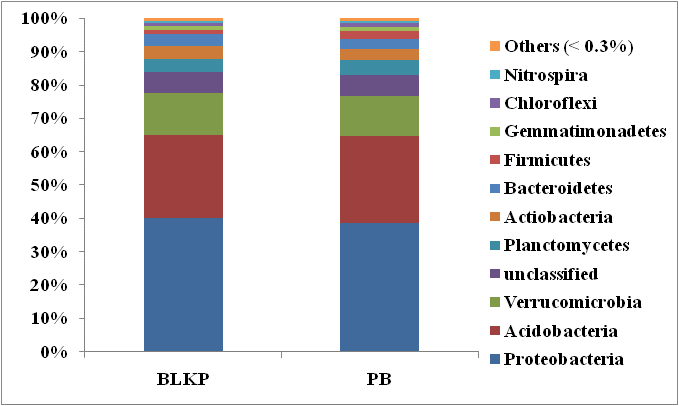


**Supplementary Figure S3 | Mean relative abundances of dominant bacterial phyla in soils from early and late successinal forests.** Phylogenetic groups accounting for less than 0.3 % of all classified sequences are summarized in the artificial group the‘others’.

**Supplementary Figure S4 | The plant diversity-productivity patterns in two temperate forest in with different successional stage in Changbai Mountain, China.** An asymptotic diversity-productivity pattern was shown in the early successional forest (PB), but the productivity does not increase with plant diversity anymore in late successional forest (BLKP).
